# Absence of an N-Linked Glycosylation Motif in the Glycoprotein of the Live-Attenuated Argentine Hemorrhagic Fever Vaccine, Candid #1, Results in Its Improper Processing, and Reduced Surface Expression

**DOI:** 10.3389/fcimb.2017.00020

**Published:** 2017-02-06

**Authors:** John T. Manning, Alexey V. Seregin, Nadezhda E. Yun, Takaaki Koma, Cheng Huang, José Barral, Juan C. de la Torre, Slobodan Paessler

**Affiliations:** ^1^Department of Pathology, University of Texas Medical BranchGalveston, TX, USA; ^2^Department of Immunology and Microbial Science, Scripps Research InstituteLa Jolla, CA, USA

**Keywords:** junin virus, vaccines, glycoproteins, glycosylation, viral proteins

## Abstract

Junin virus (JUNV), a highly pathogenic New World arenavirus, is the causative agent of Argentine hemorrhagic fever (AHF). The live-attenuated Candid #1 (Can) strain currently serves as a vaccine for at-risk populations. We have previously shown that the Can glycoprotein (GPC) gene is the primary gene responsible for attenuation in a guinea pig model of AHF. However, the mechanisms through which the GPC contributes to the attenuation of the Can strain remain unknown. A more complete understanding of the mechanisms underlying the attenuation and immunogenicity of the Can strain will potentially allow for the rational design of additional safe and novel vaccines. Here, we provide a detailed comparison of both RNA and protein expression profiles between both inter- and intra-segment chimeric JUNV recombinant clones expressing combinations of genes from the Can strain and the pathogenic Romero (Rom) strain. The recombinant viruses that express Can GPC, which were shown to be attenuated in guinea pigs, displayed different RNA levels and GPC processing patterns as determined by Northern and Western blot analyses, respectively. Analysis of recombinant viruses containing amino acid substitutions selected at different mouse brain passages during the generation of Can revealed that altered Can GPC processing was primarily due to the T168A substitution within G1, which eliminates an N-linked glycosylation motif. Incorporation of the T168A substitution in the Rom GPC resulted in a Can-like processing pattern of Rom GPC. In addition, JUNV GPCs containing T168A substitution were retained within the endoplasmic reticulum (ER) and displayed significantly lower cell surface expression than wild-type Rom GPC. Interestingly, the reversion A168T in Can GPC significantly increased GPC expression at the cell surface. Our results demonstrate that recombinant JUNV (rJUNV) expressing Can GPC display markedly different protein expression and elevated genomic RNA expression when compared to viruses expressing Rom GPC. Additionally, our findings indicate that the N-linked glycosylation motif at amino acid positions 166–168 is important for trafficking of JUNV GPC to the cell surface, and the elimination of this motif interferes with the GPC release from the ER.

## Introduction

**Figure 1:**
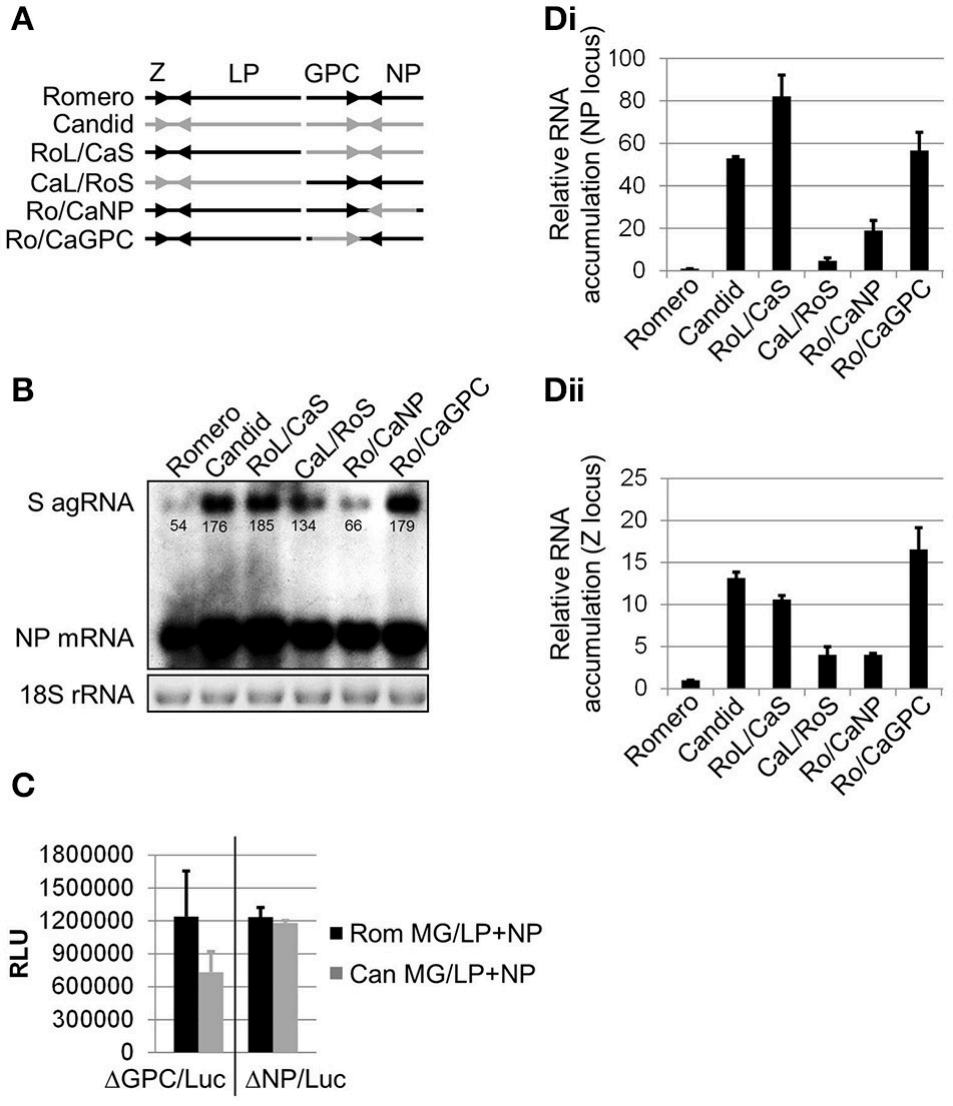
**Comparison of transcriptional activity between the polymerase complexes of chimeric JUNV variants**. **(A)** Schematic representation of rJUNV genomes. Black and gray colors indicate the genetic material of Rom and Can strains of JUNV, respectively. **(B)** Levels of anti-genomic (agRNA) S segment RNA and mRNA of the NP gene in Vero cells infected with rJUNV. Cells were infected at an MOI of 5 and harvested at 24 h p.i. Total RNA was isolated and analyzed by Northern blot for viral RNA species. Equal sample loading and RNA integrity was controlled by EtBr staining of 18S rRNA. Densitometry was calculated using AlphaEase^TM^ in order to determine saturation of each band. Band saturation of the selected area is represented on a scale from 0 to 255. **(C)** Comparison between the activities of Rom and Can LP in minigenome (MG) assays. BHK-21S cells were transfected with murine pol-I promoter-driven S segment-based minigenome (MG) constructs expressing firefly luciferase in lieu of either GPC (ΔGPC/Luc) or NP (ΔNP/Luc) gene together with pol-II expression constructs for LP and NP. The levels of luciferase expression were determined at 48 h after transfection with Rom (Rom MG/LP+NP) or Can (Can MG/LP+NP) constructs. RLU, relative light units. **(D)** Relative viral RNA accumulation transcribed from either NP **(Di)** or Z **(Dii)** gene locus. Vero cells were infected in the same conditions as in **(B)** (MOI 5) and harvested at 24 h p.i. Quantitative RT-PCR using SYBR Green dye was performed in triplicates. Levels of viral RNA normalized to GAPDH mRNA are expressed as the ratio relative to the level of viral RNA in cells infected with rRom. Average values and standard deviations are shown. Romero, rRomero. Candid, rCandid #1. RoL/CaS, rRomL/CanS. CaL/RoS, rCanL/RomS. Ro/CaNP, rRom/CanNP. Ro/CaGPC, rRom/CanGPC. Ro/CaLP, rRom/CanLP. Ro/CaZ, rRom/CanZ.

The New World arenavirus Junin (JUNV) causes Argentine hemorrhagic fever (AHF) in humans, a severe potentially lethal disease endemic to the central regions of Argentina. The virus genome consists of two single-stranded, negative sense RNA segments, small (S) and large (L). Both genome segments utilize an ambisense coding strategy to encode two viral proteins that are expressed in opposite orientation (Figure 1). The L RNA segment (~7 kb) encodes the viral RNA-dependent RNA polymerase (LP) that replicates and transcribes viral RNA genome and a small zinc-binding RING finger protein (Z) that is the arenavirus counterpart of the matrix protein found in many negative strand RNA viruses. Z has multiple functions in the viral replication cycle including formation and budding of virions from the plasma membrane, negative regulation of the activity of the viral replication complex, and modulation of the host cell response to infection (Fehling et al., 2012). The S RNA segment (~3.4 kb) encodes the glycoprotein precursor (GPC) and the nucleoprotein (NP). GPC is co- and post-translationally processed by cellular proteases to yield glycoproteins G1 and G2, and the stable signal peptide (SSP) that form G1/G2/SSP glycoprotein complexes present at the surface of mature virions and responsible for virus receptor recognition and cell entry via receptor mediated endocytosis (Burri et al., 2012). NP is the most abundantly present viral protein in both infected cells and virions. NP serves as an essential co-factor for LP transcription of RNA, and also blocks the detection of viral RNA by the cellular anti-viral defense mechanisms (Buchmeier et al., 2013).

The G2-mediated fusion between viral and cellular membranes in the acid environment of the endosome releases the viral ribonucleoprotein (vRNP) into the cell cytoplasm, which is ensued by the onset of viral RNA synthesis. Replication and expression of arenavirus genome is strictly regulated during infection. After the genetic material is delivered into the cytoplasm, the viral polymerase initiates synthesis of genomic and anti-genomic segment RNA species, as well as mRNA of viral genes. Studies of the prototypic arenavirus LCMV-infected cells demonstrated that the maximal level of viral RNA synthesis that preceded peak virus titers was followed by a marked decrease in the rate of RNA production (Cornu and de la Torre, 2001). Therefore, two phases of arenavirus replication cycle have been proposed: the active genome replication and expression phase and the virion assembly and budding phase. Z is not required for RNA synthesis mediated by vRNP but Z was shown to exhibit a dose-dependent inhibitory effect on the activity of vRNP (Cornu and de la Torre, 2001). Subsequently, studies with the New World arenavirus Machupo (MACV) demonstrate that Z forms a direct heterodimeric complex with the homologous LP and lock the LP in a promoter-bound form, thus preventing LP from copying the template genome RNA. Interestingly, the ability of Z to inhibit LP activity from heterologous arenaviruses was found to correlate with the degree of genetic distance between the viruses. Thus, the Z of a distantly related Old World arenavirus LCMV neither interacted nor inhibited the functional activity of MACV LP (Kranzusch and Whelan, 2011). This Z-mediated inhibition of vRNP-directed viral RNA synthesis has been proposed to play an important role in the regulation of the arenavirus life cycle. Thus, in the early phase of infection, low concentration of Z allows for active viral RNA synthesis; however, accumulation over time of high levels of Z in infected cells leads to the Z-LP complex being locked bound to the viral promoter, which may facilitate inclusion of the viral polymerase into arenavirus virions (Kranzusch et al., 2010; Kranzusch and Whelan, 2011).

A single amino acid change in the transmembrane region of Candid #1 (Can) G2 has been demonstrated to significantly attenuate JUNV neurovirulence in mice (Albariño et al., 2011) and in a guinea pig model that closely reproduces key features of human AHF (Emonet et al., 2011). This mutation, F427I, has been shown to destabilize the metastable conformation of the glycoprotein complex triggering transition to a fusogenic conformation at neutral pH. This could possibly limit tissue dissemination of Can (Droniou-Bonzom et al., 2011). However, the F427I mutation occurred during passages in fetal rhesus monkey lung FRhL-2 cells (Ambrosio et al., 2011) and cannot account for the attenuated phenotype associated with the Can predecessors XJ37 and XJ44 strains of JUNV (Yun et al., 2008).

Previously, the GPC of LCMV has been demonstrated to induce endoplasmic reticulum (ER) stress and trigger unfolded protein response (UPR) (Pasqual et al., 2011). Thus, acute infection with LCMV induced production of the immunoglobulin heavy-chain-binding protein (BiP), an ER-resident protein that provides initial folding assistance to completely unfolded or unstructured nascent polypeptide chains (Schroder, 2008), and selectively activated the activating transcription factor 6 (ATF6)-controlled branch of UPR while the protein kinase RNA-like ER kinase (PERK)- and the kinase/endonuclease inositol-requiring protein 1 (IRE1)-regulated pathways were neither activated nor blocked. Expression of the individual LCMV proteins demonstrated that only production of the GPC triggered ATF6-mediated UPR, observed during LCMV-infection. Importantly, rapid down regulation of GPC expression during transition from acute to persistent LCMV infection resolved ER stress conditions and returned UPR signaling to basal levels (Pasqual et al., 2011).

Here, we present evidence that cells infected with rJUNV expressing the GPC of Can exhibit higher levels of RNA synthesis and protein production than cells infected with rJUNV expressing the GPC of the pathogenic Rom strain of JUNV. We also show that Can GPC exhibits impaired processing and altered trafficking that promotes induction of the ER stress response and GPC's ability to interact with Z. Altered GPC-Z interaction can influence regulation of replication and expression of the viral genome, which may contribute to enhanced immunogenicity of Can.

## Materials and methods

### Cells and viruses

African green monkey kidney epithelial Vero, human embryonic kidney (HEK) 293, and baby hamster kidney (BHK-21) cells (American Tissue Culture Collection) were maintained in Dulbecco's modified Eagle's medium supplemented with 10% (Vero and HEK 293) and 5% (BHK-21) fetal calf serum (HyClone) and an antibiotic-antimycotic solution (Gibco). Generation and *in vitro* and *in vivo* characterization of the chimeric rJUNV used in the current study has been described previously (Emonet et al., 2011; Seregin et al., 2015). Briefly, cDNA of both genome segments of Rom (GenBank accession numbers AY619640 and AY619641) and Can (GenBank accession numbers AY746353 and AY746354) were cloned in antigenomic orientation into a plasmid vector containing a murine RNA polymerase I promoter (mPol-I) to generate mPol-I-Sag and mPol-I-Lag plasmids. The open reading frames (ORF) of the minimal viral trans-acting factors, NP and LP, required for RNA replication and transcription by the JUNV polymerase complex, were cloned into an RNA polymerase II (pol-II) expression plasmid vector to generate pC-NP and pC-LP plasmids. Chimeric rJUNV were generated by genetically engineering the ORF of Can genes into the Rom genome. rJUNV were rescued in BHK21 cells by transfection with the corresponding mPol-I-Sag and mPol-I-Lag plasmids together with the pol-II plasmids pC-NP and pC-LP expressing the NP LP of Rom. Viral stocks were prepared by infecting Vero cells [multiplicity of infection (MOI) = 0.01] and collecting virus-containing tissue culture supernatants (TCS) at 4 days post-infection (p.i.), followed by elimination of cell debris by centrifugation (10,000 g for 10 min at 4°C). Work with virulent strains of JUNV was performed in the UTMB biosafety level 4 (BSL-4) facilities in accordance with institutional health and safety guidelines.

### Northern blotting

Total RNA from infected cells lysed in TRIzol Reagent (Invitrogen) was isolated using the Direct-zol RNA MiniPrep kit (Zymo Research) following the manufacturer's protocol in triplicate. The Northern blot analysis was performed using the NorthernMax-Gly kit (Ambion). Briefly, 1 ug of total RNA was denatured in glyoxal loading buffer containing EtBr at 50°C for 30 min. RNA was separated by electrophoresis in 1% low electroendosmosis (LE) agarose gel and transferred onto the BrightStar-Plus positevily charged nylon membrane (Ambion) by downward passive transfer for 2 h. Before hybridization with RNA probes, EtBr-stained 18S rRNA was visualized under long-waive UV light and photographed to assess the quality of RNA and to ensure equal sample loading. To prepare the RNA probe for the detection of S gRNA and GPC mRNA species, a 590 nt PCR fragment was amplified from Romero mPol-I-Sag spanning positions 743–1333 of the S segment. The probe was *in vitro* transcribed using the MAXIscript T7 kit (Ambion) from the T7 promoter included in the primer annealing at position 743 and biotinylated using the BrightStar Psoralen-Biotin Kit (Ambion) according to the protocols provided by the manufacturer. The RNA probe targeting the S agRNA and NP mRNA species was synthesized from a 544 nt PCR fragment spanning positions 1870–2414 of the S segment of Romero genome (T7 promoter was included in the primer annealing at position 2414). Blots were hybridized with biotinylated RNA probes at 0.1 nM overnight at 68°C followed by one low-stringency wash for 10 min at room temperature and two high-stringency washes for 15 min at 68°C. Hybridization signals were detected using the BrightStar BioDetect Nonisotopic Detection Kit (Ambion). Northern blot band densities were quantified using AlphaEaseFC^TM^ software.

### Quantitative real-time PCR

Total RNA from JUNV-infected cells was isolated in triplicate as described for Northern blot experiments. Viral RNA quantification was performed in triplicate using the iScript One-Step RT-PCR Kit with SYBR Green (Bio-Rad) with 100 ng of total RNA. The following primers were used for detection: NP direct, GGTCCTTCAATGTCGAGCCA; NP reverse, AATCACAGGCAGGTCATGGG; Z direct, AAGTGCTGCTGGTTTGC TGA; Z reverse, TCCACCGGTACTGTGATTGTG, GAPDH-Vero direct, AGTCAACGGATTTGTCGTA; GAPDH-Vero reverse, GGGTGGAATCATACTGGAAC; GAPDH-guinea pig direct, TACGACAAGTCCCTCAAGATTG; GAPDH-guinea pig reverse, TCTGGGTGGCAGTGATGG. Melt curve analysis was performed to confirm PCR fragment specificity. Sample cycle thresholds (Ct) were normalized to the Ct values of GAPDH. Relative amounts of viral RNA in infected cells and tissues were calculated against rRomero RNA using the equation 2^−ΔCt^, where ΔCt^sample^ = (Ct^sample^ − Ct^GAPDH/sample^) − (Ct^rRomero^ − Ct^GAPDH/rRomero^) (Pfaffl, 2001).

### Minigenome luciferase assay

Minigenome (MG) luciferase reporter constructs were generated by replacing both viral genes encoded by the S segment in Rom and Can mPol-I-Sag with a firefly luciferase (Fluc) and green fluorescent protein (GFP) genes. Therefore, two reporter constructs encoding Fluc in lieu of either GPC or NP were generated for each strain of JUNV. To perform the MG luciferase assay, BHK-21 cells (seeded at 1 × 10^5^/well in an 12-well plate) were transfected in triplicate with equimolar amounts of pC-NP, pC-L, and indicated MG constructs totaling in 2 ug of plasmid DNA per well. Cells in each well were also co-transfected with an expression plasmid for Renilla luciferase (100 ng/well) in order to normalize Fluc luminescence between the samples. Fourty-eight hours later, Fluc reporter gene expression was assayed using the Dual-Glo Luciferase Assay System (Promega) according to the manufacturer's protocol.

### Western blotting

Infected or transfected cells were harvested in the Laemmli's SDS loading buffer (65.8 mM Tris-HCl, pH 6.8, 2.1% SDS, 26.3% (w/v) glycerol, 0.01% bromophenol blue, 10% β-mercaptoethanol) (Bio-Rad). The samples were boiled at 95°C for 5 min prior to fractionation by SDS-PAGE in 4–15% Mini-Protean TGX gels (Bio-Rad). Proteins were transferred to polyvinyl difluoride (PVDF) membranes using the Trans-Blot Turbo electrotransfer system (Bio-Rad) according to the manufacturer's protocol. After transfer, the membranes were washed ones in distilled water and blocked in PBS-T (10 mM sodium phosphate, 0.15 M NaCl, 0.1% Tween-20, pH 7.5) containing 5% non-fat dried milk for 1 h at room temperature. Then, the membranes were probed with primary antibodies diluted in blocking buffer (1:1000) at 4°C overnight, washed three times for 15 min with PBS-T, and incubated with a secondary antibody conjugated with the horse radish peroxidase (HRP) (Cell Signaling) in PBS-T (1:1000) for 1 h at room temperature. After another wash in PBS-T, the HRP signal was visualized by enhanced chemoluminescence (ECL) (ECL Western Blotting System, Amersham). Each Western blot was performed in triplicate, and a representative blot was presented. The primary polyclonal antibodies targeting JUNV G2 and Z were raised in rabbits against synthetic peptides corresponding to amino acid sequences GKYPNLKKPTVWRR and GASKSNQPDSSRAT (ProSci), respectively, that are completely conserved between Romero and Candid #1. The anti-NP monoclonal antibody NA05-AG12 was obtained from the Biodefense and Emerging Infections Research Resources Repository (BEI Resources). The antibodies against BiP and β-actin were purchased from Cell Signaling Technology, Inc.

### Confocal microscopy

BHK-21S cells were seeded onto NeuVitro glass coverslips (12 mm, 1.5 thickness, PDL coated) using DMEM containing 5% FBS. After 24 h, cells were transfected with CMV-driven expression plasmids expressing Romero, XJ13, XJ44, Candid, RomT168A, or CanA168T GPC. All transfections were performed using XtremeGENE-9 transfection reagent (Roche) following the manufacturer's protocol. The transfected cells were incubated for 36 h and fixed with 4% paraformaldehyde for 10 min, then permeabilized for 2 min using 0.1% Triton-X100 in PBS. The cells were stained with CytoPainter Red ER stain (Abcam) and Hoechst 33342 nuclear stain following manufacturer's protocol, then stained with an anti-JUNV GPC monoclonal mouse antibody (BE08, BEI Resources) for 1 h at room temperature followed by and AlexaFluor 488 secondary goat anti-mouse antibody (Life Technologies) for 1 h at room temperature, each at a 1:500 dilution. The coverslips were mounted onto slides using MOWIOL mounting solution and allowed to dry for 24 h. All images were captured using a Zeiss LSM 510 confocal microscope. Images presented were representative of multiple frames of view for each sample.

### Flow cytometry

HEK293 cells were seeded into 12-well plates in DMEM containing 5% FBS. After 24 h, cells were transfected in triplicate using the same conditions as described for confocal microscopy. The cells were incubated for 36 h and then placed in a single-cell suspension, followed by fixation in 4% paraformaldehyde for 10 min at room temperature. Staining was performed using an anti-JUNV GPC monoclonal mouse antibody (BE08, BEI, Resources) for 1 h at room temperature followed by an APC-conjugated goat anti-mouse secondary antibody (Life Technologies) for 1 h at room temperature, using a 1:500 dilution for each.

## Results

### Attenuated variants of JUNV exhibit higher levels of RNA synthesis in infected cells than virulent variants

We have previously described generation of recombinant JUNV (rJUNV) encoding different gene combinations of the pathogenic Rom and attenuated Can strains of the virus (Seregin et al., 2015) and identified GPC as the major viral determinant of JUNV virulence and attenuation in a guinea pig model of AHF (Seregin et al., 2015). To gain further insights into the molecular basis by which GPC determines virulence vs. attenuation of JUNV virulence *in vivo*, we examined the differential patterns of viral RNA synthesis and gene expression in cells infected with different inter- and intrasegment chimeric rJUNVs (Figure 1A). To this end, Vero cells were infected (MOI = 5) with the inter- and intrasegment chimeric JUNVs and intracellular levels of viral RNA were determined by Northern blot analysis. In order to quantify each of the bands in the Northern blot, AlphaEase^TM^ software was utilized to compare band intensity. The values listed beneath each band (Figure 1B) represent the band saturation on a scale of 0–255. Intriguingly, we determined that cells infected with rJUNVs that exhibited a virulent phenotype in guinea pigs and expressed Rom GPC (rRom, rCanL/RomS, and rRom/CanNP) accumulated lower amounts of antigenomic S segment RNA and NP mRNA than cells infected with the viruses that were attenuated in guinea pigs and expressed the GPC of Can (rCan, rRomL/CanS, and rRom/CanGPC) (Figures 1B,Di). While the rCanL/RomS virus agRNA expression was statistically lower (*p* < 0.05) than the agRNA expression in viruses that were attenuated, the difference was not as pronounced as the difference between the attenuated viruses and either rRom or rRom/CanNP (*p* < 0.05). Both rRom and rRom CanNP expressed three-fold less agRNA than the attenuated viruses. We obtained similar results for the levels of genomic S RNA and GPC mRNA (data not shown). Next, the levels of L and S segment RNA synthesis were evaluated by quantitative PCR using the Z and NP loci, respectively (qPCR). The data observed in qPCR was similar to the data obtained for the agRNA in northern blots in terms of relative RNA expression. The total levels of Z (L segment) and NP (S segment) RNA were higher in Vero cells infected with the attenuated variants of rJUNV (Figures 1Di,Dii). This indicated that the experimental data obtained by qPCR was in agreement with the data obtained in Northern blot experiments. The slight differences in relative RNA expression could likely be attributed to the fact that the qPCR is detecting both genomic RNA and mRNA, whereas the Northern blot data distinguishes between the two.

To investigate possible reasons for the observed discrepancies in levels of viral RNA synthesis, we examined the activity levels of Rom and Can vRNPs using established minigenome (MG) rescue systems based on the S segments of Rom and Can (Lee et al., 2000) where the reporter firefly luciferase marker replaced either the GPC or NP of JUNV. Surprisingly, we observed similar firefly luciferase expression with homologous LP/NP combinations of Rom and Can (Figure 1C). Together, the data indicates that in cells infected with the different rJUNVs, levels of viral RNA synthesis were not determined by activity of the core vRNP.

### Virulent and attenuated variants of rJUNV exhibit different patterns and kinetics of protein expression in infected cells

**Figure 2:**
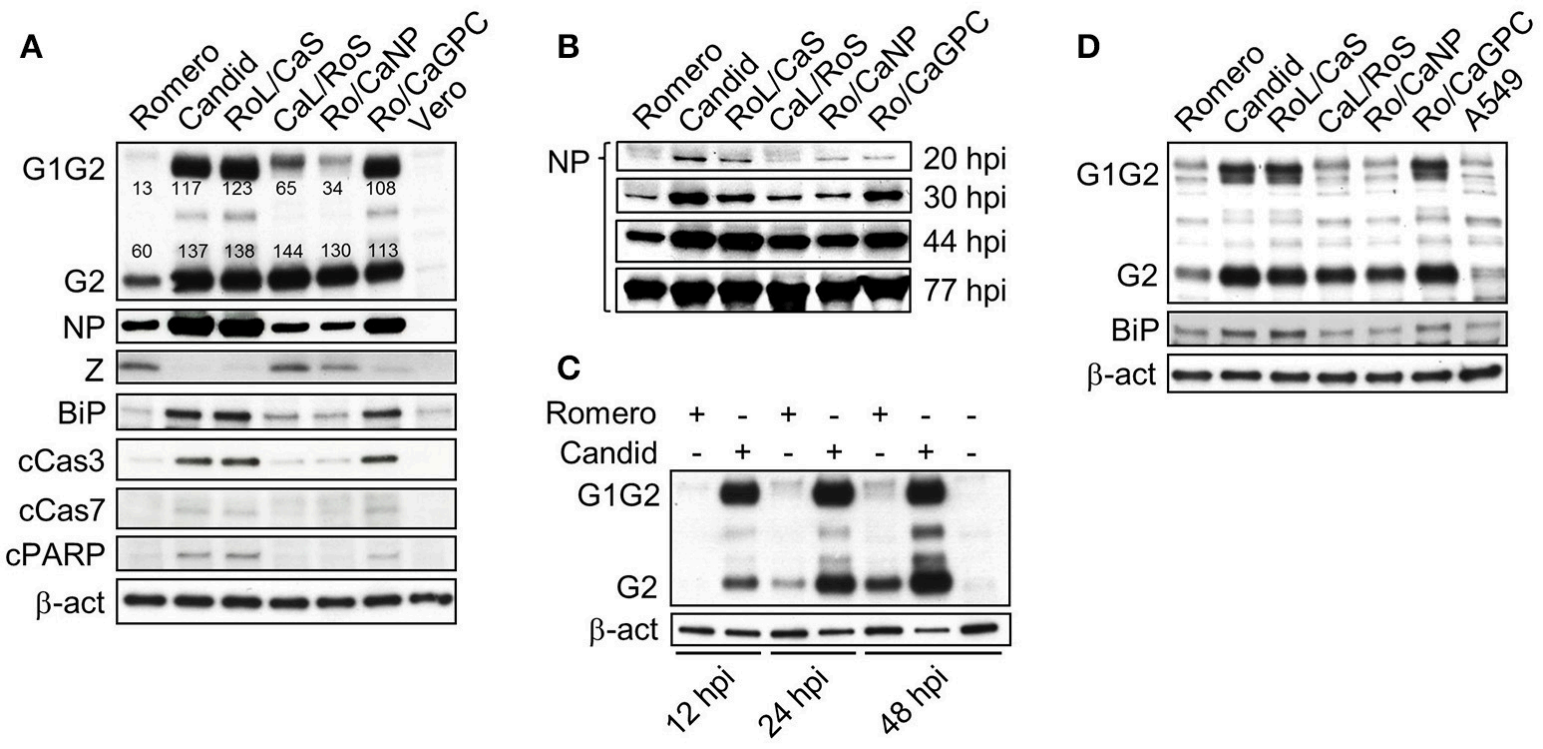
**Protein expression profiles and kinetics of chimeric JUNV variants. (A)** Differential expression of proteins by Vero cells infected at an MOI of 5. Cells were lysed in Laemmli's SDS sample buffer at 48 h p.i. Viral proteins were detected by Western blotting using antibodies raised against conserved regions of G2, NP, and Z between Rom and Can. Cellular proteins were detected using commercially available antibodies. Densitometry was calculated using AlphaEase^TM^ in order to determine saturation of each band. Band saturation of the selected area is represented on a scale from 0 to 255. **(B)** Kinetics of NP expression. Vero cells infected at an MOI of 0.1 were collected at the indicated time points and the levels of NP expression were determined by Western blotting. Equal sample loading was monitored by Comassie blue staining (not shown). **(C)** GPC expression profile and kinetics of expression between rRom (Romero) and rCan (Candid). Vero cells were infected at an MOI of 5 and cell lysates were collected at the indicated time points. Western blot was probed with an anti-G2 antibody. **(D)** Differential expression of viral GPC and the UPR marker BiP in A549 cells infected with chimeric rJUNV. Cells were infected at an MOI of 5 and harvested at 48 h p.i. The levels of viral and cellular proteins were determined by Western blotting. Romero, rRomero. Candid, rCandid #1. RoL/CaS, rRomL/CanS. CaL/RoS, rCanL/RomS. Ro/CaNP, rRom/CanNP. Ro/CaGPC, rRom/CanGPC. Ro/CaLP, Vero, lysate of mock-infected Vero cells. A549, lysate of mock-infected A549 cells.

Due to the differences in RNA expression between the rJUNVs, we elected to compare the expression patterns and kinetics of viral protein production between the rescued rJUNVs (Figure 2A). Analysis of GP expression in rJUNV-infected Vero cells revealed two distinct patterns of protein expression for rJUNVs that exhibited a virulent phenotype in guinea pigs and expressed Rom GPC and the viruses that were attenuated in guinea pigs and expressed Can GPC. The lysates of cells infected with the attenuated viruses (expressing Can GPC) exhibited differences in uncleaved G1G2 levels, which correlated with the presence of intermediate bands between G1G2 and G2 in Western blots. The proportion between the amounts of G2 and uncleaved G1G2 was notably different, as shown by densitometry quantification (AlphaEase^TM^) of the G1G2 and G2 bands for each sample. The viruses expressing Can GPC expressed a relatively equal amount of uncleaved G1G2 and cleaved G2, whereas the viruses expressing Rom GPC expressed three-fold (CaL/RoS) of four-fold (Romero, Ro/CaNP) increase in cleaved G2 over uncleaved G1G2 (Figure 2A). Additionally, there is a presence of two intermediate bands between the sizes of G1G2 and G2 in cells infected with viruses expressing Can GPC. While these bands are not present in cells infected with viruses expressing Rom GPC, it is unclear whether this is due to lower overall protein expression in these samples or the bands are indicative of a processing issue with the Can GPC (Figure 2A). Due to the additional non-specific binding of our antibody to A549 proteins, these bands are difficult to detect in A549 cells. The relative levels of G1G2 and G2 for each rJUNV are similar in A549-infected cells, indicating that the G1G2:G2 ratios observed in Vero cells are not cell-type specific (Figure 2D).

It is interesting that the total amounts of G2 and G1G2 and the amounts of NP accumulated in cells 48 h after infection were higher for the rJUNVs that were attenuated in guinea pigs, compared to the viruses that were virulent for guinea pigs (Figure 2A). Differences in NP expression levels appeared early after infection and persisted through 44 h post-infection (Figure 2B). Likewise, differences in the total GPC amounts between the virulent and attenuated rJUNV (Figure 2A) were also observed first at early times p.i., and persisted throughout the course of infection (Figure 2C). Interestingly, the presence of additional GPC bands and the increase in uncleaved G1G2 correlated with lower amounts of Z (Figure 2A), despite the observed higher RNA levels of the *Z* gene locus (Figure 1Dii). Together, these results demonstrate that the inter- and intrasegment rJUNV expressing Can GPC exhibit a distinct viral protein expression profile in infected cells that was markedly differed from that of rJUNV expressing the GPC of the virulent Rom strain, and these viruses express more protein overall with the exception of Z.

### Post-translational processing of can GPC and the ER stress response in GPC-infected and transfected cells

The inter- and intrasegment chimeric JUNVs expressing the GPC of the attenuated Can exhibited a distinct viral protein expression profile in infected cells that markedly differed from that of the viruses expressing the GPC of the virulent Rom (Figure 2A). Comparison of the GPC expression profiles in Vero and A549 cells showed that the presence of additional bands of intermediate sizes between G2 and G1G2 for rJUNV expressing the GPC of Can. The presence of these intermediate bands correlates with an increase in the presence of uncleaved G1G2 in the cell lysates, suggesting that these bands could be associated with a co- or post-translational processing issue. This was observed in both primate and human cells suggesting the involvement of co- or post-translational processing mechanisms conserved for both species. These results allowed us to hypothesize that the nascent GPC of Can undergoes abnormal processing that results in decreased production of fully processed G2, ER accumulation of abnormally processed GPCs in ER, and induction of ER stress. To examine this hypothesis we assessed expression levels of BiP in Vero and A549 cells infected with rJUNV expressing the GPC of either Can or Rom. BiP is an ER chaperone protein that senses unfolded proteins (UP) in the ER lumen and activates the cellular UP response (UPR) (Liu et al., 2003). Activation of BiP expression has previously been utilized to demonstrate UPR induction in LCMV-infected cells (Pasqual et al., 2011). Remarkably, both Vero (Figure 2A) and A549 (Figure 2B) cells exhibited an increase in BiP expression upon infection with rJUNVs expressing the Can GPC. In contrast, levels of BiP expression were only slightly elevated in cells infected with rJUNVs expressing the Rom GPC. Prolonged unresolved ER stress may result in the induction of apoptosis, which may play a role in the attenuated phenotype and immunogenicity of Can (Tabas and Ron, 2011). We therefore examined cells infected with our chimeric rJUNVs for the presence of markers associated with ER stress-induced apoptosis signaling. Cells infected with rJUNV expressing Can GPC showed higher levels of cleaved caspase 3, and a small increase in both caspase 7 and poly (ADP ribose) polymerase (PARP) over those infected with rJUNV expressing Rom GPC. This indicates that expression of Can GPC, but not Rom GPC, promotes pro-apoptotic UPR signaling in infected cells (Figure 2A).

**Figure 3:**
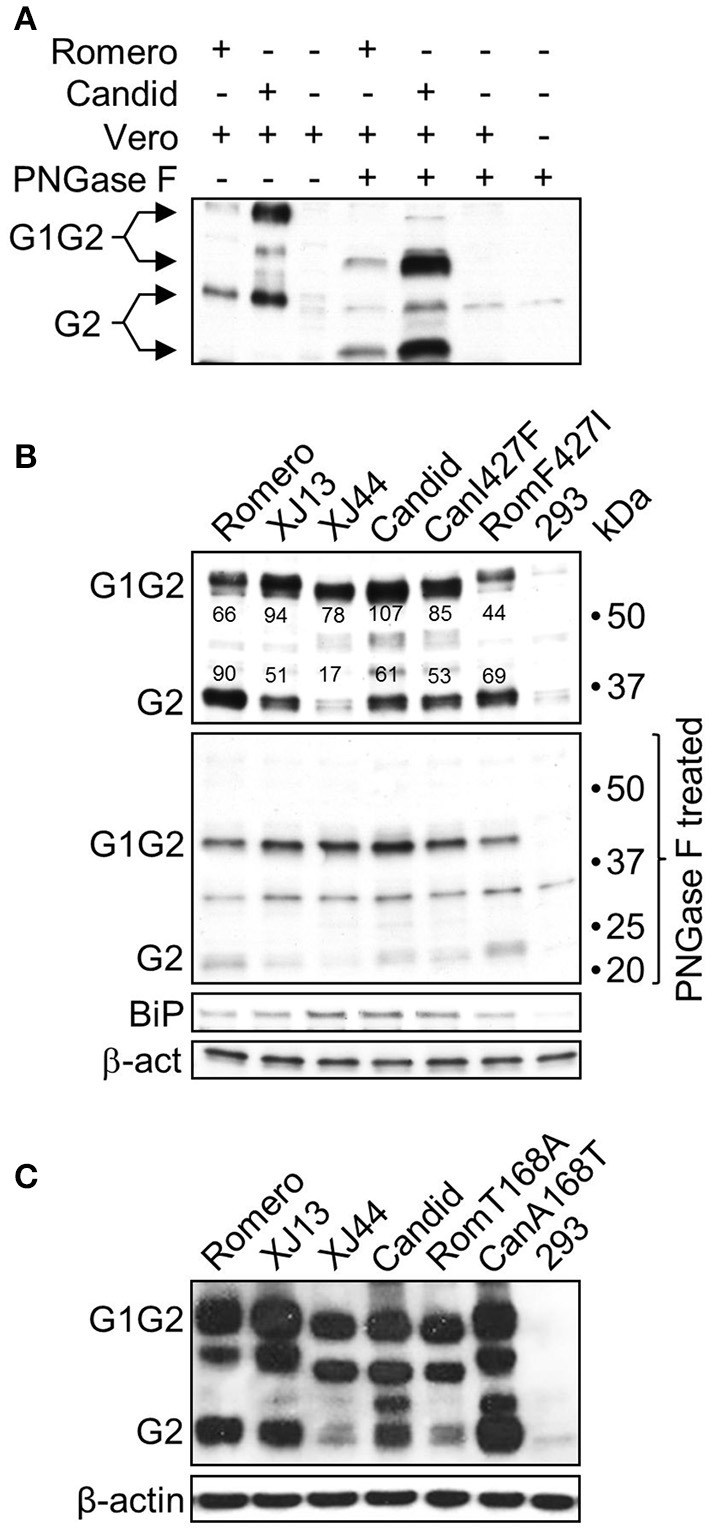
**N-linked glycosylation status of JUNV GPC. (A)** Lysates of Vero cells infected at an MOI of 5 with either rRom (Romero) or rCan (Candid) were treated with PNGase F to remove N-linked oligosaccharides from protein backbone. The GPC expression profiles were analyzed by Western blotting using an anti-G2 antibody. **(B)** Activation of the UPR in HEK 293 cells expressing GPC of JUNV strains differing in *in vivo* attenuation status. Cells were transfected with equal amounts of expression plasmids for the GPC of Rom (Romero), Can (Candid), Can predecessor strains XJ13 and XJ44, and the GPC of Can and Rom bearing I427F (CanI427F) and F427I (RomF427I) amino acid changes in the G2 subunit, and A168T and T168A amino acid changes in the G1 subunit **(C)**, respectively. Cell lysates were harvested at 24 h post-transfection and divided into two equal aliquots that were either mock-treated (top panels) or treated with PNGase F (PNGase F treated). Expression of viral GPC and the UPR marker BiP was analyzed by Western blotting. 293, HEK 293 cells transfected with a GFP expressing construct that shares the same plasmid backbone with the expression constructs for JUNV GPC. Densitometry was calculated using AlphaEase^TM^ in order to determine saturation of each band. Band saturation of the selected area is represented on a scale from 0 to 255. **(C)** GPC expression profiles in cells with or without the T168A substitution. Cells were transfected with equal amounts of each plasmid and allowed to incubate for 36 h. HEK 293 cell were transfected with a GFP plasmid containing the same backbone as the GPC expression plasmids. The cells were lysed and analyzed by Western blotting.

To further investigate whether the appearance of additional GPC species detected by Western blot in cells infected with rJUNV expressing Can GPC was due to abnormal post-translational modification of Can GPCs, we treated lysates of infected Vero cells with the peptide-N-glycosidase F (PNGase F) to remove N-linked glycans from the protein backbone. Treatment with PNGase F shifted the protein bands of intermediate size observed in untreated samples into a single intermediate band (Figure 3A). The anti-G2 antibody we used for Western blot exhibited a high degree of non-specific binding to PNGase F; however, this band had a higher mobility than the GPC bands in untreated samples and did not completely mask the bands between untreated G2 and G1G2 (Figure 3A). These results indicated that the observed difference in the ratios of uncleaved G1G2 to cleaved G2 between Rom and Can and the associated presence of intermediate bands with the accumulation of G1G2 were likely caused by accumulation of glycosylation intermediates created in ER processing.

Can was generated by serial *in vivo* and *in vitro* passaging of a virus isolate from a clinical human case of AHF (Parodi et al., 1958). During this process the virus gradually lost its virulence for guinea pigs, non-human primates, mice, and humans (Albariño et al., 2011). To investigate whether the attenuation of Can correlates with the efficiency of GPC processing and post-translational modifications, we generated expression constructs for Rom and Can GPC, as well as the two Can predecessors XJ13 and XJ44. We also generated constructs encoding the Rom GPC containing the F427I mutation within the G2 transmembrane region and the Can GPC containing the reverse I427F mutation to test whether this amino acid position that was critical for JUNV neurovirulence in mice (Albariño et al., 2011) and virulence in guinea pigs (Seregin et al., 2015) would also play a role in processing and post-translational glycosylation of GPCs. To this end, we transfected human embryonic kidney (HEK) 293 cells with the different GPC constructs and examined the differences in G1G2 to G2 ratios and differences in intermediate band expression, as well as BiP expression, by Western blot (Figure 3B). Remarkably, the processing efficiency and glycosylation pattern of GPCs correlated with the mutations accumulated during the transition from Rom to Can GPCs (note the appearance of additional bands and increased mobility of the G1G2 precursor). To quantify this difference, densitometry was performed using AlphaEase^TM^. The analysis revealed that there is roughly 50% more cleaved G2 than uncleaved G1G2. This shifts to a 2:1 ratio of uncleaved G1G2 to cleaved G2 in XJ13, a 4:1 ratio of uncleaved G1G2 to cleaved G2 in XJ44, and a 2:1 ratio of uncleaved G1G2 to cleaved G2 in Can (Figure 3B). As expected, PNGase F treatment resulted in the disappearance of the intermediate bands into one single band. In addition, BiP expression levels also slightly increased in XJ44 and Can-transfected cells even during plasmid overexpression. Interestingly, the presence of F or I residues at position 427 neither affected post-translational processing or glycosylation of either of the GPCs, nor changed the level of BiP expression. Collectively, these results demonstrated a correlation between the accumulation of uncleaved G1G2 and the degree of JUNV attenuation *in vivo*. The slight increase in BiP differs from the substantial increase observed in infection. This could be due to the effects of plasmid overexpression of the protein, which leads to more rapid expression and accumulation of the protein than what would be experienced during infection.

The increase in uncleaved G1G2 begins as early as XJ13 GPC, and these changes become more pronounced in cells transfected with XJ44 GPC (Figure 3B). Compared to the highly pathogenic Rom strain, XJ13 contains three amino acid substitutions, while only one single additional amino acid substitution that destroys a glycosylation motif at N166 occurred between XJ13 and XJ44. This single mutation (T168A) in G1 region correlates with impaired GPC cleavage (Figure 3B). To confirm that the T168A substitution is sufficient to cause the observed impaired processing of XJ44 and Can GPCs, we incorporated this mutation into the Rom GPC expression construct, and the A168T reversion into the Can GPC expression construct. Western blot analysis revealed that the T168A mutation in Rom GPC resulted in a similar G1G2 to G2 ratio to the one observed for XJ44 GPC, with the lysate containing predominantly uncleaved G1G2. The A168T reversion increased the processing efficiency of GPC, yielding an increase in cleaved G2 over what is observed in Can GPC-transfected cells (Figure 3C). GPCs containing the mutation T168A, predicted to abrogate N-linked glycosylation at N166, exhibited faster electrophoretic migration. This strongly suggests the absence of an N-linked glycan at N166 that is otherwise present in Rom GPC. It is interesting that the intermediate bands begin to appear even in the Rom GPC-transfected samples in a plasmid overexpression systems at later time points (36 h post-transfection as opposed to 24 h post-transfection) where the protein is expressed at higher levels than encountered in infection (Figure 3C). This indicates that accumulation of uncleaved G1G2 potentially contributes to the presence of the intermediate bands regardless of the presence of the glycan at N166. Together, these results demonstrate that the T168A mutation is a major determinant of the decrease in G1/G2 cleavage in Can GPC. The presence of the intermediate bands could be indicative of a processing issue and could associate with an increase in BiP, and both the intermediate bands and BiP upregulation appear in an overexpression system regardless of the GPC expressed at later time points.

### Glycoproteins of attenuated strains of JUNV accumulate in the endoplasmic reticulum of infected cells

**Figure 4:**
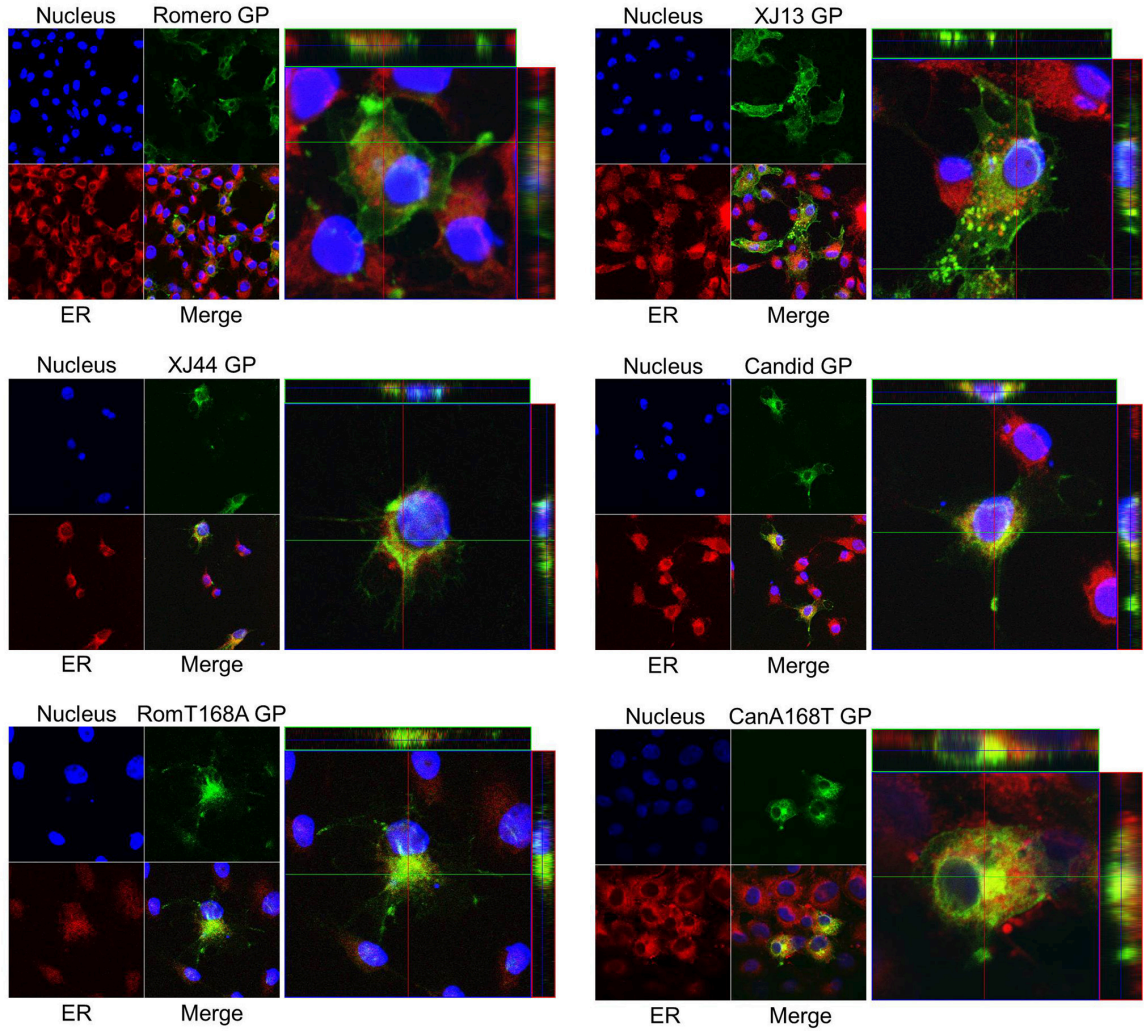
**Intracellular localization of JUNV GPC relative to the ER and cell surface**. Monolayers of HEK293 cells were transfected with CMV-driven expression plasmids expressing Romero, XJ13, XJ44, Candid, Romero expressing the T168A substitution (RomT168A), and Candid expressing the A168T reversion (CanA168T). At 36 h post-transfection, cells were permeabilized and stained with both an ER-selective fluorescent dye (red) and Hoechst 33342 nuclear dye (blue), and an anti-JUNV GPC primary antibody followed by a conjugated AlexaFluor^TM^ secondary antibody (green). Cells were imaged using a Zeiss LSM 510 confocal microscope. Both the merged images on a single Z plane (left) and a cross-section of a single representative cell sectioned along along the Z axis (right) are included.

The increase in uncleaved G1G2 in XJ13, XJ44, Can, and Rom-T168A GPC-expressing cells with respect to cleaved G2 amounts led us to hypothesize that the uncleaved G1G2 was accumulating in the ER. The cleavage of G1G2 by SKI-1/S1P occurs in the late Golgi, suggesting that the protein is not reaching the Golgi for transport to the cell surface. This ratio of uncleaved G1G2 to cleaved G2 is largest in XJ44 and Rom-T168A GPC, and is restored partially in Can GPC. XJ44 was passed 19 times in FRhL cells to generate the seed stocks for Can. During this time, four additional amino acid substitutions occurred in GPC (Albarino et al., 2009), which may have resulted in partial rescue of GPC processing (Figure 3). We therefore examined whether GPC could efficiently traffic from the ER to the late Golgi where GPC cleavage occurs, or if the protein was co-localizing with the ER through the use of confocal microscopy. At 24 h post-transfection, the majority of Rom GPCs accumulated on the cell surface, while much lower concentrations remained in the ER. In contrast, XJ13 GPs were evenly distributed between the ER and the cell surface. Interestingly, GPs appeared to accumulate within vesicles in cells expressing XJ13 GPC (Figure 4). While the identity of these vesicles is unclear, we presume that they are part of the Golgi apparatus. The XJ44, Can and RomT168A GPCs predicted to lack the N-linked glycan at N166 co-localized mostly with the ER, and restoration of the glycosylation motif in Can GPC (CanA168T) resulted in a larger concentration of GPs at the cell surface. Notably, patterns of subcellular distribution for the different GPs correlated with their corresponding GPC cleavage patterns (Figure 3). These results indicated that mutation T168A, predicted to eliminate the N-linked glycan at N166, plays an important role in ER retention of Can GPC, preventing Can GPs from reaching the cell surface.

### Glycoproteins of virulent Rom, but not attenuated XJ13, XJ44, and can are efficiently expressed on cell surface

**Figure 5:**
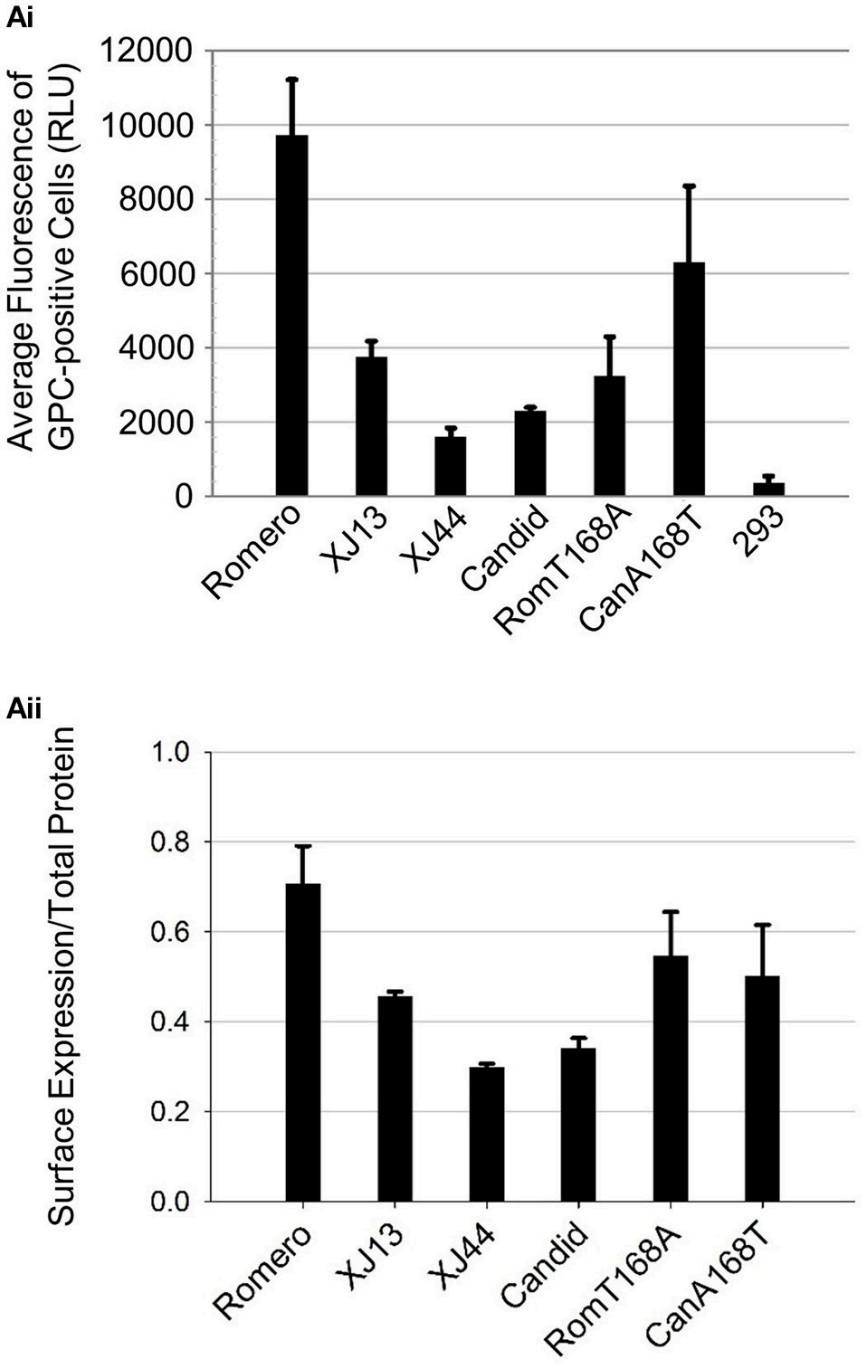
**Cell surface expression of JUNV GPC**. Monolayers of HEK293 cells were transfected with CMV-driven expression plasmids previously used in triplicate (Figure 4). At 24 h post-transfection, the cells were re-suspended and fixed with 4% paraformaldehyde. The cells were split into two separate aliquots per sample, and one aliquot was permeabilized with Triton-X100 while the other was left untreated. The re-suspended cells were stained with an anti-JUNP GPC primary antibody and subsequently stained with a secondary antibody conjugated with APC. Suspended cells were subjected to flow cytometry. **(Ai)** Bar graph representing the average intensity of fluorescence-positive cells that were Triton-X100-untreated, expressed in fluorescence units obtained from individual cells. Fluorescence-positive cells were gated based on the fluorescence of the mock-transfected HEK293 control group. Average intensity values of the three separate experiments (bars) and standard error among the triplicates (lines) are both shown. **(Aii)** Bar graph representing the percentage of cells expressed on the cell surface. The aliquots treated with Triton-X100 were stained in the same manner as **(Ai)** and the average fluorescence intensities were measured through cell sorting. The results from **(Ai)** were divided by the obtained values for the total protein. Average percentages (bars, expressed as decimal values) and standard error among triplicates are both shown.

Since the mutation T168A presumably caused the absence of the N-linked glycan at N166 and the retention of GPs in the ER, we hypothesized that JUNV GPs containing mutation T168A would exhibit decreased cell surface expression. To test this hypothesis, we used flow cytometry to quantify levels of GPs at the cell surface for each of our GPC constructs (Figure S1). We split the samples into two separate aliquots, permeabilizing only one of the aliquots. The non-permeabilized samples allowed us to quantify GPs present on the cell surface (Figure 5Ai). Cells transfected with Rom GPC expressed the highest levels of GPs at the cell surface, and we observed a declined gradient in GPC cell surface expression from XJ13 to XJ44 and Can GPC transfected cells. Additionally, we observed a large decreased in GP surface expression when the G1 N166 glycosylation motif was removed from Rom GPC. Likewise, we were able to rescue Can GP surface expression by restoring the T residue at position 168 (Figure 5Ai). We calculated the percentage of GPC expressed on the cell surface with respect to the total GPC from permeabilized cells and compared the samples. Both XJ44 and Can GPC show a decrease in surface expression percentage compared to Rom GPC (Figure 5Aii). These results indicated that Rom GPCs efficiently reach the cell surface, while a steady decline in cell surface expression is observed in XJ13, XJ44, and Can GPC transfected cells. Expression of GPCs containing the T168A mutation resulted in only a small fraction of GPCs reaching the cell surface with respect to Rom GPCs. However, the decrease in RomT168A surface expression is more directly linked to a lower total protein expression despite equal plasmid transfection.

## Discussion

The mechanisms responsible for the attenuation of the Can strain of JUNV, which is currently used as a live-attenuated vaccine in AHF-endemic regions of Argentina (Ambrosio et al., 2011), remain to be elucidated. Previous studies have demonstrated that a single amino acid change (F427I) in the transmembrane region of the G2 subunit of the envelope glycoprotein complex significantly attenuates neurovirulence of JUNV in mice (Albariño et al., 2011). This single substitution has been proposed to destabilize the metastable conformation of the glycoprotein complex, favoring its transition to the fusogenic conformation at neutral pH and possibly limiting the dissemination of the virus (Droniou-Bonzom et al., 2011). However, intraperitoneal inoculation of guinea pigs with rRom containing the F427I substitution resulted in a self-limiting acute illness, suggesting that mutation F427I in the G2 can only partially account for the highly attenuated phenotype of Can *in vivo* (Seregin et al., 2015). Moreover, the mutation F427I was fixed in the JUNV genome during the final stages of the generation of Can vaccine during passages in FRhL-2 cells, and after the virus had already achieved attenuation in guinea pigs and non-human primates (Ambrosio et al., 2011). This further indicates that other mutations in GPC are also important for Can attenuation. Consistent with these findings, only rJUNV expressing the full-length GPC of Can were completely attenuated and did not induce any detectable disease symptoms in the guinea pig model of AHF (Seregin et al., 2015). Interestingly, the Can glycoprotein complex has shown an increased dependence on the TfR-1 for target cell entry (Droniou-Bonzom et al., 2011), and the receptor-binding domain has been mapped to the G1 subunit (Nunberg and York, 2012; Buchmeier et al., 2013). Therefore, the role of other molecular mechanisms than those associated with the F427I amino acid change in G2 in attenuation of JUNV would have to be investigated. Among the additional mutations is a single amino acid substitution that occurred between XJ13 and XJ44 (T168A), which eliminates an N-linked glycosylation motif present in G1 (Albarino et al., 2009).

Can and Rom GP exhibited distinctly different G1G2 to G2 ratios that were detected by Western, as well as differences in the efficiency of GPC processing characterized primarily by an increase in uncleaved G1G2 and the associated presence of intermediate bands in the Can GPCs (Figure 2). Treatment with PNGase F prior to Western blot analysis resulted in detection only of GPC and G2 species and the collapse of the two intermediate bands into a single band (Figure 3), supporting that the intermediate GP bands correspond with an increase in uncleaved G1G2, and contain various glycosylation profiles. Interestingly, the presence of the intermediate bands do not correlate with the presence of specific mutations, but rather the accumulation of uncleaved G1G2 in the cells. This is supported by the presence of an intermediate band created by Rom GPC during plasmid overexpression for 36 h. At this later time point, even the Rom GPC began to accumulate uncleaved G1G2 within the transfected cells despite maintaining its cleaved G2 levels. The faster migration of GPC from both XJ44 and Can (Figure 3) correlated with the presence of the mutation T168A that is predicted to remove an N-linked glycosylation motif from N166-T168. The abnormal glycosylation profile of XJ44 and Can could result in the accumulation of misfolded proteins in the ER, which could trigger the ER stress cellular response. Consistent with this possibility we observed increased expression levels of BiP in cells infected with viruses expressing Can GPC (Figure 2A). However, cells transfected with either Rom or Can GPC do not exhibit as pronounced of a difference in BiP expression. We believe that this is due to plasmid over-expression in these cells, where both proteins are expressed at levels much higher than what is observed during infection, leading to similar BiP expression. The ER chaperone BiP is the primary detector of ER stress, and is responsible for the activation of the three main branches of the UPR through the mediators IRE1, PERK, and ATF6 (Schroder, 2008). Transient upregulation of BiP was also found in LCMV-infected cells (Pasqual et al., 2011). Sustained UPR due to failure to timely resolve accumulation of misfolded proteins within the ER, will trigger cell apoptosis (Schroder, 2008), a situation that has been described in a number of virus infections (Dimcheff et al., 2004; Medigeshi et al., 2007; Barry et al., 2010). Apoptosis has been described to occur more frequently in Can-infected cells than in Rom-infected cells (Kolokoltsova et al., 2014), a finding consistent with our results documenting increased levels of BiP and cleaved caspases (Figure 2). The correlation between attenuation and UPR-associated pro-apoptotic signaling indicates that the improper processing of Can GPC could contribute to a protective immune response through the upregulation of pro-inflammatory genes (Zhang and Kaufman, 2008).

The mutation T168A is predicted to destroy an N-linked glycosylation motif from N166-T168. This mutation arose prior to XJ44 and correlated with severe impaired GPC processing. During subsequent passages of XJ44 in FRhL cells to generate Can, the virus acquired four additional amino acid substitutions (Ambrosio et al., 2011), leading to a partial rescue of GPC cleavage and trafficking of GPs to the cell surface (Figure 3C). We therefore suspected that the mutation T168A was the primary contributor to the impaired and abnormal processing of Can GPC. The results of the incorporation of the T168A and A168T substitutions into plasmids expressing Rom and Can GPC, respectively, support this hypothesis (Figure 3C). We observed that combined expression of all GP species derived from GPC containing 168A was lower than the one observed for GPC containing 168T. Based on evidence of the activation of the UPR in cells expressing GPC deficient of the glycosylation motif, we suspect that the cause of the decrease in overall GP could be due to ER-associated degradation (ERAD) triggered by the abnormal glycosylation of GPC 168A (Vembar and Brodsky, 2008).

Both XJ44 and Can GPC exhibited impaired GPC processing compared to Rom GPC (Figure 3). Cleavage of GPC into G1 and G2 by SKI-1/S1P does not occur until GPC reaches the Golgi (Lenz et al., 2001; Beyer et al., 2003). Confocal microscopy revealed that GPC containing mutation T168A co-localized mostly with ER markers in transfected cells, supporting the hypothesis that GPC cleavage was impaired due to ER retention of GPC. While we cannot rule out the possibility that GPC fails to be cleaved due to an inherent folding issue that makes the cleavage motif inaccessible, Western blot analysis indicated that a fraction of Can GPC was still cleaved (Figures 2, 3). Furthermore, the ratios of uncleaved G1G2 to cleaved GPC present in Western blot analysis (Figure 3B) are in rough correlation with the fractions of total GPC present on the cell surface for the Rom, XJ13, XJ44, and Can (Figure 5Aii). Although we describe a significant difference in protein expression between Rom and Can GPC at the cell surface, the GPC concentration required to successfully infect target cells *in vitro* remains unclear since the Rom and Can viruses display no significant differences in infectious particle production in cell culture (Seregin et al., 2015). The decrease in GPC concentration at the cell surface would likely affect the concentration of GPC on virus particles, and while this does not seem to affect the infectivity in cell culture, there could be a significant effect on infectivity of the virus *in vivo*. An LCMV strain with a modified G1G2 cleavage site (Clone 13^FURIN^) has previously been shown to be attenuated despite similar growth *in vitro* (Popkin et al., 2011). Both the Rom GPC bearing the single T168A substitution and XJ13 GPC expressed intermediate levels of GP at the cell surface when compared to Rom and XJ44 GPC, indicating that other amino acid substitutions present in XJ13 also contributed to the incorrect trafficking of XJ13 GPs to the cell surface. Interestingly, XJ13 GP appears to accumulate in small vesicles between the ER and the cell surface. While the identity of these vesicles has not been elucidated, we suspect them to be Golgi vesicles. There are additional N-linked glycosylation trimming steps and quality controls which take place in the Golgi (Arvan et al., 2002), and it is possible that one or both of the amino acid substitutions present in the XJ13 G1 affected the processing and sorting of XJ13 GPs during Golgi quality control.

Reduced expression levels of Can GP complex at the cell surface (Figure 5) might have contributed to reduced intracellular levels of Z protein in infected cells that express Can GPC (Figure 2A). The Z protein interacts with the GP complex to mediate its incorporation into the budding virion (Capul et al., 2007; Schlie et al., 2010). It has been shown that budding of JUNV Z from the cell does not require the presence of the other viral proteins (Perez et al., 2003). Therefore, it is possible that the Z protein is budding independently of the Can GP complex. This hypothesis is supported by the fact that the release of Z from the cell is inhibited by the co-expression of GPC (Groseth et al., 2010). This phenomenon of independent Z budding could also help to explain the differences observed in RNA expression between the rJUNVs. The increase in RNA in the viruses that are attenuated also correlates strongly with an absence of Z in the infected cells. The Z protein is an inhibitor of the LP, and an absence of the Z protein would lead to a higher polymerase activity in the infected cells.

Collectively, our findings support the conclusion that Can GPC induces ER stress through improper post-translational processing and retention of the viral GP within the ER. The mechanism of GP retention within the ER and how this contributes to the attenuation of the live vaccine have yet to be elucidated.

## Author contributions

JM and AS contributed equally to the conception and design of the experiments, and share first authorship of the manuscript. NY assisted in the rescue and propagation of all recombinant viruses, and the collection of RNA and cell lysates for both Western and Northern blotting. TK assisted with the Flow Cytometry. NY, CH, JB, TK, Jd, and SP assisted JM and AS with data analysis and the interpretation of results.

### Conflict of interest statement

The authors declare that the research was conducted in the absence of any commercial or financial relationships that could be construed as a potential conflict of interest.

## Supplementary material

Figure S1 **Flow cytometry quantification of surface and total cellular GPC**. HEK293 cells were transfected with CMV-driven GPC expression plasmids in triplicate. At 24 h post-transfection, the cells were re-suspended and fixed with 4% paraformaldehyde. The cells were split into two separate aliquots per sample, and one aliquot was permeabilized with Triton-X100 while the other was left untreated. The re-suspended cells were stained with an anti-JUNP GPC primary antibody and subsequently stained with a secondary antibody conjugated with APC. Suspended cells were subjected to flow cytometry. **(Ai)**. Fluorescence intensities of each cell without treatment with Triton-X100, representing surface GPC expression. The bar represents the population of positive cells. **(Aii)**. Fluorescence intensities of each cell with treatment with Triton-X100, representing total cellular GPC. The bar represents the population of positive cells.
