# Supplementary figures and images for: Absence of an N-Linked Glycosylation Motif in the Glycoprotein of the Live-Attenuated Argentine Hemorrhagic Fever Vaccine, Candid #1, Results in Its Improper Processing, and Reduced Surface Expression
Source: Front Cell Infect Microbiol. 2017 Feb 6;7:20. doi: 10.3389/fcimb.2017.00020 (PMC5292626; doi:10.3389/fcimb.2017.00020)

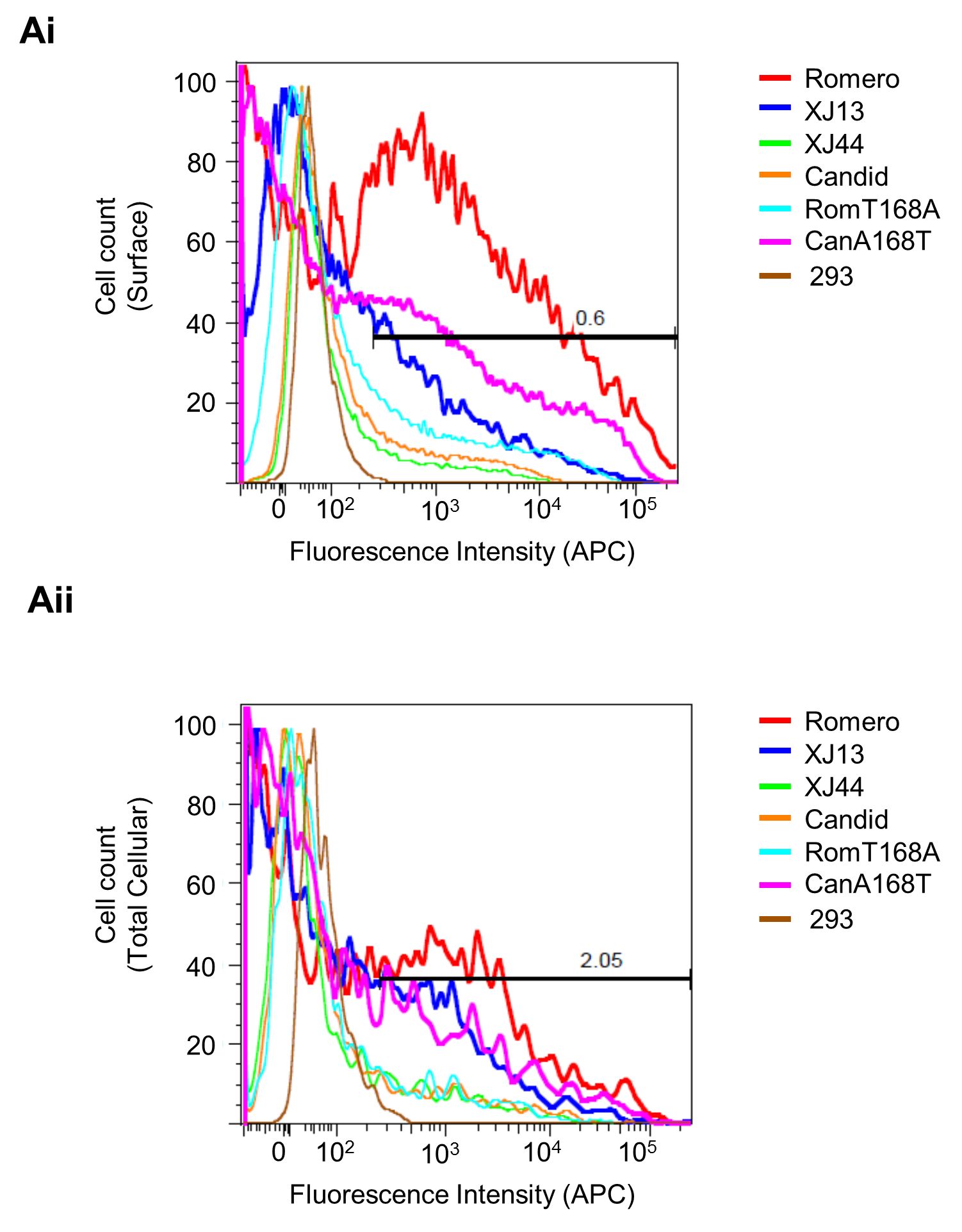

Supplement: Figure S1 — Flow cytometry quantification of surface and total cellular GPC. HEK293 cells were transfected with CMV-driven GPC expression plasmids in triplicate. At 24 h post-transfection, the cells were re-suspended and fixed with 4% paraformaldehyde. The cells were split into two separate aliquots per sample, and one aliquot was permeabilized with Triton-X100 while the other was left untreated. The re-suspended cells were stained with an anti-JUNP GPC primary antibody and subsequently stained with a secondary antibody conjugated with APC. Suspended cells were subjected to flow cytometry. (Ai). Fluorescence intensities of each cell without treatment with Triton-X100, representing surface GPC expression. The bar represents the population of positive cells. (Aii). Fluorescence intensities of each cell with treatment with Triton-X100, representing total cellular GPC. The bar represents the population of positive cells. [file Image1.JPEG]
